# Risk factors for anemia in patients with chronic kidney disease: A protocol for systematic review and meta-analysis

**DOI:** 10.1097/MD.0000000000027371

**Published:** 2021-10-08

**Authors:** Ping Yin, Quan Wu, Lihong Shou, Xiaohui Dong

**Affiliations:** a Department of Hematology, Huzhou Central Hospital, Affiliated Cent Hospital of Huzhou University, No. 1558, Sanhuanbei Road, Wuxing District, Huzhou, Zhejiang, PR China; b Clinical Laboratory, Huzhou Central Hospital, Affiliated Cent Hospital of Huzhou University, No. 1558, Sanhuanbei Road, Wuxing District, Huzhou, Zhejiang, PR China.

**Keywords:** adverse effects, anemia, chronic kidney disease, meta-analysis

## Abstract

**Background:**

Anemia in patients with chronic kidney disease (CKD) presents significant impacts on patients, the health-care system, and financial resources. Therefore, this study aimed to identify the risk factors of anemia among CKD patients.

**Methods:**

This meta-analysis was conducted in accordance with the guidelines of the Preferred Reporting Items for Systematic Reviews and Meta-Analyses Protocols statement guidelines. Studies were identified through systematic searches in September 2021 with no restrictions on date and time, and publication status using the following bibliographic databases: Embase, Medline, PubMed, Web of Science, Science Direct, and the Cochrane Library. The search was conducted using the following terms and phrases: “anemia”, “risk factors”, “associated factors”, “chronic kidney injury”, “chronic kidney disease”, and “chronic renal insufficiency”. The quality of each included study was assessed according to the Newcastle-Ottawa scale. Meta-analysis was performed using STATA^TM^ version 14 statistical software for Windows^TM^.

**Results:**

The results of this systematic review and meta-analysis will be published in a peer-reviewed journal.

**Conclusion:**

This meta-analysis may help policymakers and program managers design evidence-based interventions on preventing the occurrence of anemia with CKD patient populations.

## 1 Introduction

Chronic kidney disease (CKD) is an important public health problem that has become a global health concern.^[1–3]^ CKD is defined as objective renal damage for at least 3 months or lowering the glomerular filtration rate below 60 mL/min/1.73 m^2^.^[4]^ It is emerging as a complex global health problem with a huge economic burden both on the affected family of patients and on the healthcare delivery system. Although different from one country to another, the global prevalence of CKD is 242 in a million with an annual increase of 8%.^[5]^ With the growing world population, an increasing number of end-stage renal disease patients can be also predicted. Such patients are largely managed using peritoneal dialysis, or hemodialysis.^[6]^

Anemia is a serious complication of CKD and has significant adverse outcomes. Despite many advances in understanding of anemia, effective and safe treatment strategies are limited, and a large proportion of CKD patients with anemia still do not meet the hemoglobin targets. When diseased kidney loses its ability to produce the erythropoietin essential to the production of hemoglobin, anemia is developed.^[7,8]^ Although the primary cause of anemia in patients with CKD is the inadequate production of erythropoietin by the kidneys to support erythropoiesis, there is also the result of a complex interplay between patient-specific attributes including diabetes with or without nephropathy, advanced CKD stages, nutritional deficiency (iron, folic acid, and vitamin B12), diabetes mellitus, hematological disorders, not taking iron supplements, respiratory disorders, body mass index <18.5 kg/m^2^, history of hemodialysis and rural residence, smoking, and reduced serum albumin.^[9,10]^

Different primary studies worldwide have shown the risk factors of anemia as a health issue for patients with CKD. However, variation was observed among these studies. Therefore, this protocol for systematic review and meta-analysis aimed to identify risk factors for anemia in patients with CKD.

## 2 Methods

This meta-analysis was registered at Open Science Framework registries (registration number: 10.17605/OSF.IO/SUHR7) and was conducted in accordance with the guidelines of the Preferred Reporting Items for Systematic Reviews and Meta-Analyses Protocols statement guidelines.^[11]^ Ethics application was not required as this study is based on published trials.

### 2.1 Search strategy

Studies were identified through systematic searches in September 2021 with no restrictions on date and time, and publication status using the following bibliographic databases: Embase, Medline, PubMed, Web of Science, Science Direct, and the Cochrane Library. The search was conducted using the following terms and phrases: “anemia”, “risk factors”, “associated factors”, “chronic kidney injury”, “chronic kidney disease”, and “chronic renal insufficiency”. Boolean operators such as “AND” and “OR” were used to combine search terms. The reference lists of the included studies were also checked for additional studies that were not identified with the database search.

### 2.2 Eligibility criteria

Studies were included in the meta-analysis if they fulfilled the following criteria: all observational studies investigating risk factors of anemia in patients with CKD, articles published in peer reviewed journals or grey literature, and articles published in English or Chinese from inception to 2021. Studies were excluded if: they were not fully accessible, they were duplicated citations, and they possessed a poor quality score as per the stated criteria.

### 2.3 Data extraction and quality assessment

Two independent investigators screened the titles and abstracts of all potential studies. Data were extracted from each of these studies using the standardized data extraction format prepared in a Microsoft Excel worksheet by the 3 authors independently. For each included article, we extracted data regarding the name(s) of the author(s), year of publication, study area, study design, sample size, data collection year, sampling technique, diagnostic criteria used for anemia, reported prevalence with its 95% confidence interval, and information regarding the associated factors.

The quality of each included study was assessed using the Newcastle-Ottawa scale.^[12]^ Items assessed included selection of cases/cohorts and controls, comparability of study design and analysis, outcome assessment and adequacy of follow-up. ‘Stars’ were allocated for each item included in the Newcastle-Ottawa Quality Assessment Scale for a quantitative appraisal of overall quality of the individual studies. A maximum of 9 stars can be allocated to any 1 study. A study was considered to have a low risk of bias if it was allocated the maximum number of stars. A median score of 6 stars was used to distinguish moderate-and high-quality studies from poorer quality studies. The evidence grade was assessed using the guidelines of the GRADE (Grading of Recommendations, Assessment, Development, and Evaluation) working group including the following items: risk of bias, inconsistency, indirectness, imprecision, and publication bias. GRADE pro Version 3.6 software (USA) is used for the evidence synthesis.

### 2.4 Statistical analysis

To obtain risk factors for anemia in patients with CKD, a meta-analysis using the random-effects DerSimonian and Laird model was performed.^[13]^ Cochran Q chi-square statistics and I^2^ statistical tests were conducted to assess the random variations between primary studies. To investigate the sources of heterogeneity, meta-regression and subgroup analyses were performed. Potential publication bias was assessed by visually inspecting funnel plots and objectively using the Egger bias test. Sensitivity analysis was used to see the effect of a single study on the overall effect estimation. Meta-analysis was performed using STATA^TM^ version 14 statistical software for Windows^TM^.

## 3 Discussion

This study aimed to synthesize evidence on the risk factors of anemia in patients with CKD at a global level. Anemia is a well-known complicating feature of CKD and typically correlates directly with the degree of kidney impairment. According to the Kidney Disease Improving Global Outcomes Anemia Work Group, anemia in CKD occurs when the hemoglobin level is <13 g/dL for men and <12 g/dL for women.^[14]^ The potential adverse clinical outcomes of anemia in CKD patients include: cognitive impairment, angina, cardio-renal anemia syndrome, left ventricular hypertrophy, higher healthcare costs and reduced quality of life, increased hospital admission rate, worsening CKD, accelerated progression of heart disease, and increased mortality.^[15]^ Some studies have shown that early identification and prompt treatment of anemia through near normalization of hemoglobin and iron levels in CKD patients is associated with improved health-related quality of life.^[16]^ In addition, optimizing the hematocrit value before initiating dialysis may reduce mortality.^[17]^

This study has clinical implications in that the high magnitude of anemia in patients with CKD should guide healthcare professionals to minimize the risk of anemia by providing guidance to the patient who could be detected in health checkups, give information about possible risk factors during routine patient care, and provide knowledge about potential risk of anemia. This meta-analysis may help policymakers and program managers design evidence-based interventions on preventing the occurrence of anemia with CKD patient populations.

## Author contributions

Ping Yin: writes the protocol; Quan Wu: data analysis; Lihong Shou: data collection; Xiaohui Dong: study design.

**Conceptualization:** Quan Wu.

**Data curation:** Lihong Shou.

**Funding acquisition:** Xiaohui Dong.

**Writing – original draft:** Ping Yin.

**Writing – review & editing:** Xiaohui Dong.
